# A process-driven platform to manage datasets for research

**DOI:** 10.23889/ijpds.v1i1.292

**Published:** 2017-04-19

**Authors:** Gordon McAllister

**Affiliations:** University of Dundee

## Objectives

- Accumulate, manage and control shared access to research data
- Transform and maintain transformation state information about research data
- Analyse and investigate data in related sets using open and bespoke tools
- Publish extracted data to a secure safe haven environment

## Approach

The Research Data Management Platform (RDMP) is a set of data structures and processes, sharing a core Catalogue, to manage electronic health records, genomic data and imaging data throughout their lifecycle from identification and acquisition to safe disposal or archival and retention in secured Safe Havens (SH).

The architecture components of the RDMP consist of the Catalogue and five internal processes: Data Load, Catalogue Management, Data Quality, Data Summary, and Data Extraction. These are designed to enforce rigorous information governance standards relevant to the processing and anonymisation of personal identifiable data. The Catalogue serves as the single ‘source of truth’ about the datasets which all RDMP processes consult. This facilitates repeatable, reliable and auditable operations on the data.

The novelty of the RDMP is that it dynamically and seamlessly captures and preserves data transformation processes along with the primary research data to promote reuse and curation of continuously accruing research data repositories in a secure SH environment. Thus, the RDMP brings transparency and reproducibility that benefits research programmes in a way that archival of static data objects does not.

## Results

The RDMP has been in production use since July 1st 2014. There are 107 datasets configured in the Catalogue, with up to 67 dataset extractions for each of 48 research projects. It has provided data for 32 high-impact journal papers published in the last year.

Improvements in turnaround time:

- Research project data provision reduced from six months to two weeks
- Data loading reduced from two days to a few hours
- Research query response reduced from days to within a day, due to improved and standardised metadata catalogue

## Conclusion

The RDMP is a key component in automating the regular release of datasets and rationalising dataset changes over time to ensure reliable delivery of extracts to research projects. The tools and processes comprising the RDMP not only fulfil the RDM requirements of researchers, but also support seamless collaboration of data cleaning, data transformation, data summarisation and data quality assessment activities by different research groups.

